# Phenotypic plasticity of four Chenopodiaceae species with contrasting saline–sodic tolerance in response to increased salinity–sodicity

**DOI:** 10.1002/ece3.4515

**Published:** 2019-02-10

**Authors:** Yingxin Huang, Gaohua Fan, Daowei Zhou, Jiayin Pang

**Affiliations:** ^1^ Northeast Institute of Geography and Agroecology Chinese Academy of Sciences Changchun China; ^2^ School of Agriculture and Environment The University of Western Australia Perth Western Australia Australia; ^3^ The UWA Institute of Agriculture The University of Western Australia Perth Western Australia Australia

**Keywords:** biomass allocation, growth traits, phenotypic plasticity, saline–sodic soil, tolerance

## Abstract

It is unknown whether phenotypic plasticity in fitness‐related traits is associated with salinity–sodicity tolerance. This study compared growth and allocation phenotypic plasticity in two species with low salinity–sodicity tolerance (*Chenopodium acuminatum* and *C. stenophyllum*) and two species with high salinity–sodicity tolerance (*Suaeda glauca* and *S. salsa*) in a pot experiment in the Songnen grassland, China. While the species with low tolerance had higher growth and allocation plasticity than the highly tolerant species, the highly tolerant species only adjusted their growth traits and maintained higher fitness (e.g., plant height and total biomass) in response to increased soil salinity–sodicity, with low biomass allocation plasticity. Most plasticity is “apparent” plasticity (ontogenetic change), and only a few traits, for example, plant height:stem diameter ratio and root:shoot biomass ratio, represent “real” plasticity (real change in response to the environment). Our results show that phenotypic plasticity was negatively correlated with saline–sodic tolerance and could be used as an index of species sensitivity to soil salinity–sodicity.

## 1 INTRODUCTION

Environmental variation in space and time affects the distribution of species and components of the community. Plants can not only rapidly grow under resource‐rich conditions but also colonize resource‐poor habitats due to phenotypic plasticity in response to varying environments (Andersen, Mayor, & Turner, 2017; Garzon, Alia, Robson, & Zavala, 2011; van Kleunen & Fischer, 2005; Schlichting, 1986). Phenotypic plasticity is defined as the ability of an organism, with its singular genotype, to express a range of phenotypes depending on the environmental conditions (Bradshaw, 1965; Schlichting, 1986). Phenotypic plasticity, including the adjustment of morphological traits and physiological activities, may minimize the deleterious effects of the environment, thus maximizing the survival, growth, and reproduction of plants (Nicotra et al., 2010; Weiner, 2004). Phenotypic plasticity has therefore been used to estimate the potential distributional ranges of species (Valladares et al., 2014). High phenotypic plasticity, that is, the rapid change of phenotypic traits in response to environmental change, might result in the invasion success of exotic species and species replacement during succession (Bhattarai et al., 2017; Huang, Zhao, Zhou, Zhang, & Zheng, 2012; Niinemets, Valladares, & Ceulemans, 2003; Pigliucci, 2005). Interestingly, the phenotypic plasticity of fitness‐related traits was found to be negatively associated with species tolerance to environmental stress (e.g., shading, flooding, and drought stress), and therefore, phenotypic plasticity has been used as an index of species sensitivity to environmental stress (Chmura, Modrzyński, Chmielarz, & Tjoelker, 2017; Couso & Fernández, 2012; Huang et al., 2015; Poorter, Niinemets, Poorter, Wright, & Villar, 2009; Portsmuth & Niinemets, 2007; Valladares, Wright, Lasso, Kitajima, & Pearcy, 2000). In contrast, several studies have shown that the phenotypic plasticity of highly tolerant species might be similar to or higher than that of species with low tolerance (e.g., shade‐tolerant vs. shade‐intolerant species) (Kitajima, 1994; Valladares & Niinemets, 2008). It is worth noting that congeneric species are ideal materials for comparing plant plasticity, but it is difficult to find congeneric species with contrasting tolerance to abiotic stress. Therefore, dominant species are often used as an alternative (Chmura et al., 2017; Valladares et al., 2002).

When responding to biotic or abiotic environmental variation, plants often change in terms of their phenotypic and physiological activity, ultimately adjusting their growth and biomass allocation. Biomass distribution and the adjustment of growth characteristics are key factors determining the growth rate and performance of species under different environmental conditions (van Hees, 1997; Jarčuška, 2009). However, evidence suggests that growth traits are more sensitive to the environment than biomass allocation (Curt, Coll, Prevosto, Balandier, & Kunstler, 2005), which is mostly ontogenetic and varies little with environmental availability (Reich, Tjoelker, Walters, Vanderklein, & Bushena, 1998). Plasticity caused by ontogenetic shifts under varying environmental conditions is referred to as “apparent” plasticity, while plasticity that involves variation in environmental conditions that affects the rate of plant development is referred to as “real” plasticity (Weiner, 2004). The understanding of “apparent” and “real” plasticity would help in the investigation of the difference between growth and biomass allocation plasticity.

Over half a billion hectares of the land surface area around the world has been salinized (Chartres, 1993; Gupta & Abrol, 1990; Rengasamy & Olsson, 1991; Wang et al., 2010). This soil salinity–sodicity restricts the distribution of plant species, and no species can establish under the most extreme conditions of salinity–sodicity. Due to long‐term overgrazing and the reclamation of natural grassland, secondary bare saline–sodic patches are very common on the Songnen Plain, China, resulting in low community diversity, with many plant communities consisting of single species, for example, *Suaeda glauca*,* S. salsa*, or *Chloris virgata* (Jiang, He, Wu, & Zhou, 2010; Zheng & Li, 1999). So far, most studies comparing phenotypic plasticity between tolerant and intolerant species have focused on shading, flooding, and drought stress (Couso & Fernández, 2012; Lazarus, Richards, Gordon, Oki, & Barnes, 2011; Valladares et al., 2005), and there have been very limited efforts addressing saline–sodic stress. To our knowledge, only one study has compared the phenotypic plasticity of early and late successional congeneric species in response to saline–sodic soil (Huang et al., 2015); therefore, plant plasticity in response to saline–sodic stress is still largely unknown.

In this study, we aimed to evaluate plasticity in growth traits and biomass allocation between species with low and high saline–sodic tolerance over a salinity–sodicity gradient in the Songnen grassland of northern China. This study includes four plant species from the family Chenopodiaceae: two species with low saline–sodic tolerance (*Chenopodium acuminatum* Willd. and *Chenopodium stenophyllum* Koidz.) and the other two with high saline–sodic tolerance (*Suaeda glauca* (Bunge) Bunge. and *Suaeda salsa* (L.) Pall.), as found previously (Guan, Lin, Zhou, & Yu, 2013; Ma, Lv, Li, & Liang, 2014; Yang, Shi, & Wang, 2008). The following questions were addressed: (a) Does plasticity differ between high and low saline–sodic tolerant species? (b) Are the growth traits more sensitive than biomass allocation in response to salinity–sodicity? and (c) Is the plasticity largely ontogenetic?

## 2 MATERIALS AND METHODS

### 2.1 Plant species and growth conditions

Four plant species, including two saline–sodic sensitive species (*Chenopodium acuminatum* and *C. stenophyllum*) and two saline–sodic tolerant species (*Suaeda glauca* and *S. salsa*) from the same family, were included in this study. All four species are annuals found in the Songnen grassland, with *C. acuminatum* and *C. stenophyllum* often found in soils with low saline–sodic levels, while *S. glauca* and *S. salsa* dominate soils with high saline–sodic levels (Guan et al., 2013; Li & Yang, 2004). Seeds of all four species were collected from wild plants growing in the Songnen grassland in 2011.

The field trial was conducted in 2012 at the Changling Ecological Research Station for Grassland Farming (ERSGF), Chinese Academy of Sciences (44°33′ N, 123°31′ E, 145 m a.s.l.), located in the southern Songnen grassland, China. This region has warm, humid summers and cold, dry winters, with a mean temperature of 23°C in July and −20°C in January. The mean annual rainfall is 410 mm, with 80% occurring between July and September.

Plastic pots (25 cm in diameter × 25 cm in depth) were buried in the field, with the top edge of the pots approximately 1 cm above the ground. Thirty‐to‐forty seeds per pot were sown on 4 May 2012, and thinned to one seedling per pot at the two‐leaf stage 2 weeks after sowing. To eliminate positional effects and avoid potential contamination from leachate, each treatment combination as a group was rotated clockwise every 2 weeks; meanwhile, the pots within each treatment were also rotated randomly. All pots were well watered throughout the experiment.

The experiment was a two‐factorial (four species, five salinity–sodicity levels) completely randomized design. There were five salinity–sodicity treatments, including non‐, low, moderate, high, and extreme saline–sodic levels, as shown in Table 1, with 30 replicates, giving a total of 600 pots. Soil was taken from the top 30 cm of five different patches at the natural grassland of ERSGF, with a radius of 7–8 m for each patch. To minimize the large variation in soil salinity–sodicity, all five patches were located within 1 km of one another. Soils taken from each patch were air‐dried, passed through a 4‐mm sieve, and then mixed thoroughly. Information on the soil nitrogen, pH, electric conductivity, and exchangeable sodium percentage is shown in Table 1.

**Table 1:** Soil chemical properties and dominant vegetation in four saline–sodic soil treatments. Data are means ± *SE* of three measurements

Soil treatments	Nitrogen content (mg/g)	pH	Electric conductivity (dS/m)	Exchangeable sodium percentage (%)	Dominant species
Nonsaline–sodic soil	1.097 ± 0.050 a	8.41 ± 0.03 e	0.062 ± 0.001 b	1.23 ± 0.94 b	*C. glaucum* and *C. acuminatum* *
Low saline–sodic soil	1.031 ± 0.019 a	8.95 ± 0.09 d	0.097 ± 0.003 b	6.42 ± 0.40 b	*C. glaucum* †
Moderate saline–sodic soil	0.973 ± 0.045 a	9.77 ± 0.03 c	0.118 ± 0.002 b	12.08 ± 0.62 b	*L. chinensis*
High saline–sodic soil	1.231 ± 0.008 a	10.19 ± 0.02 b	0.264 ± 0.001 b	15.43 ± 0.38 b	*Chloris virgata*,* S. glauca and S. salsa*
Extreme saline–sodic soil	0.178 ± 0.003 b	10.79 ± 0.01 a	2.386 ± 0.003 a	77.26 ± 4.83 a	No species

The different letters are not statistically significant in columns.

* The abandoned cropland.

† In the ecotone between dunes and grassland.

### 2.2 Plant measurements

Twenty intact plants from each treatment per species were destructively harvested on 20 August 2012. The roots were washed carefully, and the plants were separated into roots, stems, leaves, and reproductive organs. All samples were dried in an oven at 80°C for 48 hr, and the dry weights were recorded.

Based on previous studies, fitness‐related traits (including growth and allocation traits) were analyzed to evaluate the phenotypic plasticity of the species showing contrasting tolerance to salinity–sodicity (Javid, Ford, & Nicolas, 2012; Kuehny & Morales, 1998; Portsmuth & Niinemets, 2007; Shi & Sheng, 2005). Growth traits, including plant height (absolute height), total biomass, stem diameter (basal stem diameter), root length (taproot length), and root diameter (taproot diameter), were measured. The ratios of plant height:stem diameter, plant height:root length, and root length:root diameter were calculated. The dry biomass of the root, leaf and stem, total shoot (including leaves, stems, and reproductive tissues), nonstem (including roots, leaves, and reproductive tissues), and nonleaf (including roots, stems, and reproductive organs) components were measured. Allocation traits, including root mass ratio (root/total biomass), stem mass ratio (stem/total biomass), leaf mass ratio (leaf/total biomass), shoot:root mass ratio (shoot/root biomass), and root:leaf mass ratio (root/leaf biomass), were calculated.

To evaluate the phenotypic plasticity of growth and biomass allocation traits, the phenotypic plasticity index was calculated using the following equation (Valladares, Sanchez‐Gomez, & Zavala, 2006; Valladares et al., 2000):

$$\text{PI}=\frac{\max T-\min T}{\max T}$$

where PI is the phenotypic plasticity index and *T* is the trait mean values in each soil treatment.

### 2.3 Statistical analysis

To overcome the assumptions of normality and homoskedasticity, permutation multivariate analysis of variance (PerMANOVA, vegan package in R software, number of permuted data sets = 999) and permutation analysis of variance (PerANOVA, lmPerm package in R software) were performed to test the main effects of species and saline–sodic treatment and their interaction on plant traits (Anderson, 2001). Species and saline–sodic treatment were treated as fixed factors. Differences among the mean values of plant traits and differences in plasticity among the species were determined using the least significant difference (LSD) tests. A *t*‐test at *p *=* *0.05 was performed to examine the difference between growth and allocation plasticity.

Allometric analysis was conducted for all calculated traits (ratio of two traits). Nevertheless, the allometric analysis for both root mass ratio and shoot:root ratio is the comparison between the shoot mass and root mass. The allometric relationship was described by log *y *= *b *+ *a* ×* *log *x*, where *a* is the scaling exponent (slope) and *b* is the allometric coefficient or “scaling factor” (*y*‐intercept). Differences in shifts of the slope and in the elevation of slopes (*y*‐intercept) among the different soil treatments were assessed using standardized major axis regression (SMA, also known as reduced major axis, RMA, SMATR package in R software) (Falster, Warton, & Wright, 2006; Warton, Wright, Falster, & Westoby, 2006). SMA analyses are appropriate for summarizing the relationship between two variables in terms of a single slope (Wright, Reich, Cornelissen, Falster, Groom, et al., 2005). In SMATR, the heterogeneity between SMA slopes is tested via a permutation test. Differences in SMA slope, elevation (intercept), and plant size (i.e., a shift along the common slope) were estimated (Wright, Reich, Cornelissen, Falster, Garnier, et al., 2005).

In the extreme saline–sodic soil, the two *Chenopodium* species did not germinate, while the two *Suaeda* species survived; thus, the data from the extreme saline–sodic soil treatment were not analyzed, and the data are shown in the Supporting Information (Table S1).

## 3 RESULTS

### 3.1 Plant trait response

Plant height, total biomass, stem diameter, root length, and root diameter significantly decreased with an increasing level of soil salinity–sodicity (Table 2 and Supporting Information Table S1). A significant interaction between species and soil salinity–sodicity was found for plant height (*p *=* *0.005), stem diameter (*p *=* *0.012), root length (*p *<* *0.001), and root diameter (*p *<* *0.001). The species with low tolerance (*C. acuminatum* and *C. stenophyllum*) had a higher reduction in their response to soil salinity–sodicity than the highly tolerant species (*S. glauca* and *S. salsa*) (Table 2 and Supporting Information Table S1). For example, stem diameter decreased by 50% for the species with low tolerance and by 20–40% for the highly tolerant species. Root diameter decreased by 60% for the species with low tolerance and 10–40% for the highly tolerant species. The plant height:stem diameter ratio differed among species based on the average across all treatments (*p *<* *0.001), with *C. stenophyllum* having the highest height:stem diameter ratio, followed by *S. glauca* and *C. acuminatum*, while *S. salsa* had the lowest value. Similarly, the root length:diameter ratio also differed among species. No significant species × soil salinity–sodicity two‐way interaction was found for either height:stem diameter ratio or root length:diameter ratio (*p *>* *0.05). No effects of species or soil salinity–sodicity were found for the plant height:root length ratio.

**Table 2:** PerANOVA for plant traits of four species in saline–sodic soils

	Species	Saline–sodic	Species × saline–sodic
*df*	1	3	3
Growth traits			
Multivariate F	58.52***	24.96***	1.92
Plant height	4.04 *	89.03***	7.97 **
Total biomass	2.68	76.77***	2.47
Stem diameter	22.98***	125.76***	6.32 *
Root length	14.36***	66.23***	11.47***
Root diameter	14.72***	99.52***	12.05***
Plant height:stem diameter ratio	61.03***	0.17	0.18
Plant height:root length ratio	0.00	2.04	0.03
Root length:diameter ratio	56.03***	3.46	0.87
Allocation traits			
Multivariate *F*	51.15***	8.21***	13.48***
Root mass ratio	12.93***	11.13***	15.75***
Leaf mass ratio	631.43***	3.86	0.02
Stem mass ratio	228.72***	10.78 **	10.05**
Shoot:root mass ratio	14.65***	6.17*	24.59***
Root:leaf mass ratio	32.69***	9.35**	9.79**

*F*‐values are presented. *, *p *<* *0.05; **, *p *<* *0.01; ***, *p *<* *0.001.

A significant interaction between species and soil salinity–sodicity was found for the root mass ratio (*p *<* *0.001), stem mass ratio (*p *=* *0.002), shoot:root mass ratio (*p *<* *0.001), and root:leaf mass ratio (*p *=* *0.002). For example, with increasing soil salinity–sodicity, low‐tolerance species showed a higher increase in shoot:root mass ratio and a higher decrease in root allocation and root:leaf mass ratio than those of high‐tolerance species. The leaf mass ratio differed significantly among species (*p* < 0.001). No effect of salinity–sodicity or two‐way interaction was found for the leaf mass ratio (Tables 2 and Supporting Information Table S1).

### 3.2 Plasticity of growth and allocation traits

**Figure 1:**
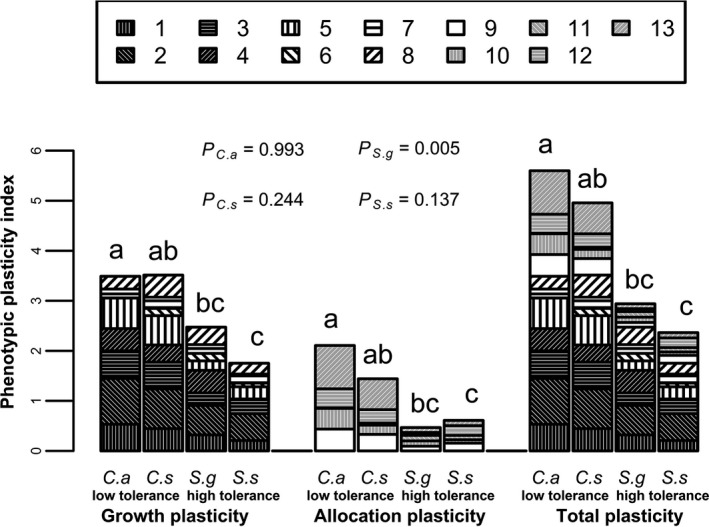
Phenotypic plasticity in four species. The four species include *C.a*:* Chenopodium acuminatum*,* C.s*:* Chenopodium stenophyllum*,* S.g*:* Suaeda glauca*, and *S.s: Suaeda* salsa. Growth traits include the following: 1: plant height, 2: total biomass, 3: stem diameter, 4: root length, 5: root diameter, 6: plant height:stem diameter ratio, 7: plant height:root length ratio, and 8: root length:diameter ratio. Structural traits include the following: 9: root mass ratio, 10: leaf mass ratio, 11: stem mass ratio, 12: shoot:root mass ratio, and 13: root:leaf mass ratio. For each compartment, bars sharing the same letters are not significantly different at *p *=* *0.05 for the four species. *p* Values were determined using a *t*‐test for mean differences between growth and allocation plasticity

The species with low tolerance (*C. acuminatum* and *C. stenophyllum*) exhibited higher growth, allocation, and total plasticity compared with the highly tolerant species (*S. glauca* and *S. salsa*) (Figure 1). No difference was found in growth, allocation, or total plasticity between the species with similar tolerance. For *S. glauca*, the growth plasticity was significantly higher than the allocation plasticity (*p *=* *0.005), while no such difference was found for other three species (*p *>* *0.05) (Figure 1).

### 3.3 Allometry of growth and allocation traits

**Figure 2:**
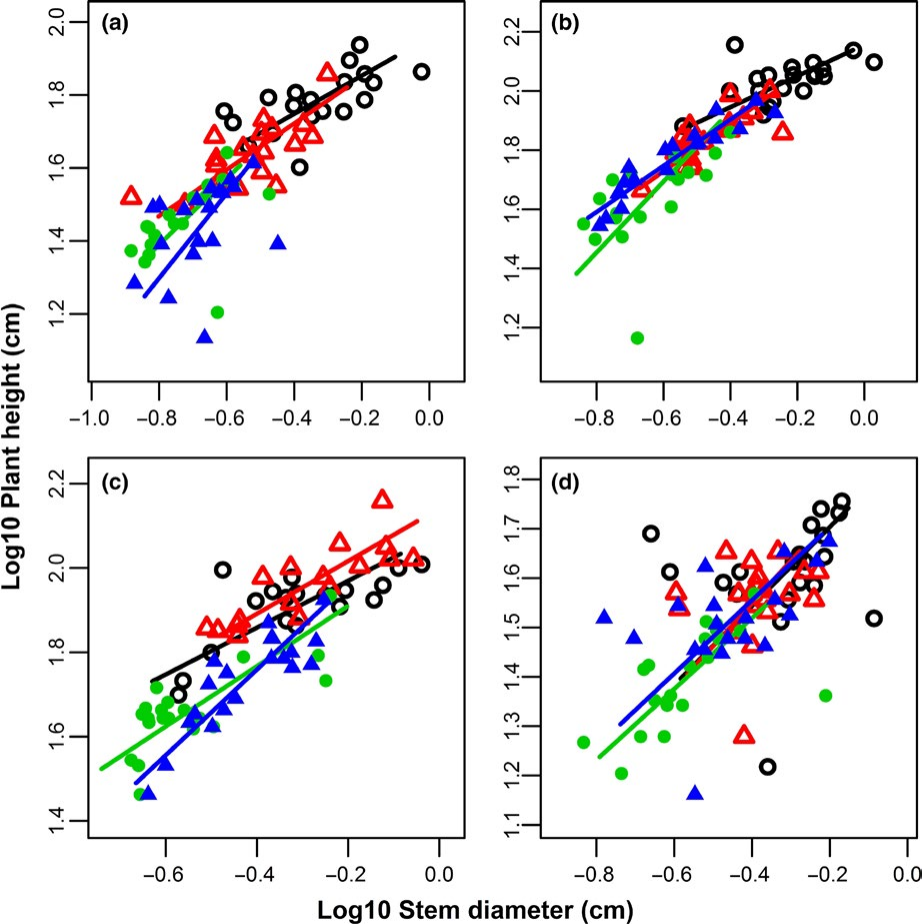
Log10–log10 plots showing the relationship between plant height and stem diameter for (a) *Chenopodium acuminatum*, (b) *Chenopodium stenophyllum*, (c) *Suaeda glauca*, and (d) *Suaeda salsa*. In each panel, the black (open circle), red (open triangle), green (filled circle), and blue (filled triangle) points and SMA fit line represent the treatments of non‐, light, moderate, and high saline–sodic soil, respectively

**Figure 3:**
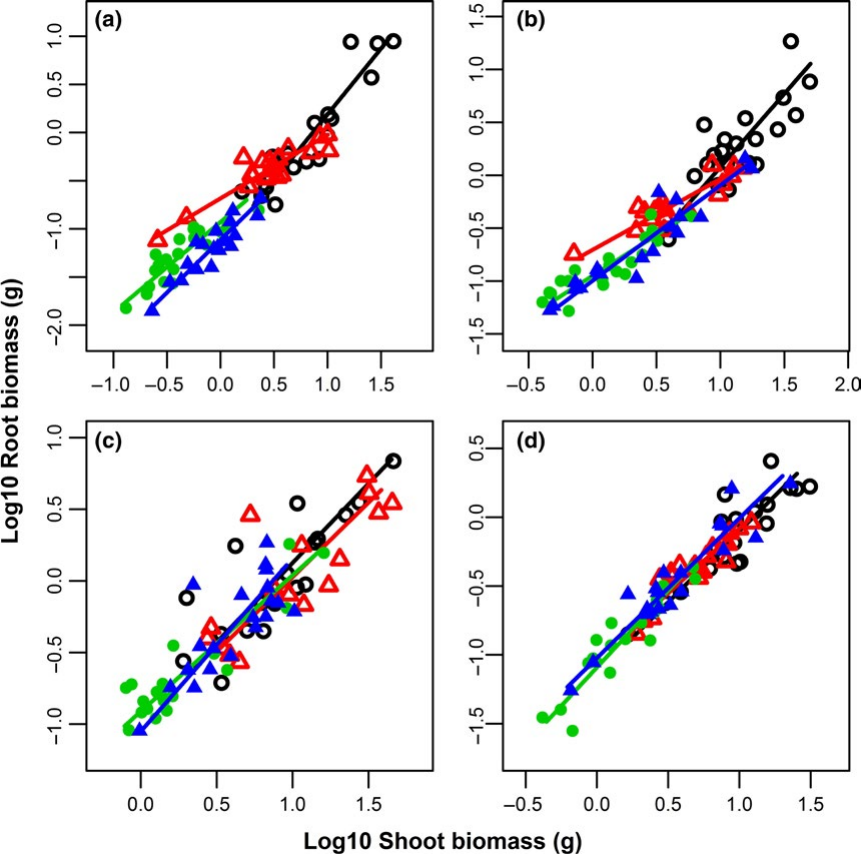
Log10–log10 plots showing the relationship between root and shoot biomass for (a) *Chenopodium acuminatum*, (b) *Chenopodium stenophyllum*, (c) *Suaeda glauca*, and (d) *Suaeda salsa*. In each panel, the black (open circle), red (open triangle), green (filled circle), and blue (filled triangle) points and SMA fit line represent the treatments of non‐, light, moderate, and high saline–sodic soil, respectively

Significant positive relationships were found between all paired traits in all species except the relationship between plant height and root length in *S. salsa* and between root length and root diameter in *S. glauca* (Table 3). The slope homogeneity of the pairwise comparisons was significant for plant height vs. stem diameter for *C. acuminatum*,* C. stenophyllum*, and *S. glauca*, plant height vs. root length for *C. stenophyllum*, root biomass vs. shoot biomass for *C. acuminatum* and *C. stenophyllum*, and root biomass vs. leaf biomass for *C. stenophyllum*, while there was no significant difference for the other pairwise comparisons. However, there were significant shifts in elevation in 20 pairwise comparisons, and there were significant shifts along a common SMA slope for all pairwise comparisons (Table 3). For *C. acuminatum*,* C. stenophyllum*, and *S. glauca*, the allometric scaling slope of plant height vs. stem diameter increased with an increasing level of soil salinity–sodicity (Table 3; Figure 2), and the differences among the different saline–sodic treatments were significant for all three species (all *p *<* *0.05 for slope homogeneity), but not for *S. salsa* (*p *>* *0.05 for both slope homogeneity and shift in elevation). For *C. acuminatum* and *C. stenophyllum*, the allometric scaling slope of root biomass vs. shoot biomass decreased with increasing soil salinity–sodicity (Table 3; Figure 3), and the differences among the different saline–sodic soil treatments were significant (*p *<* *0.001 and *p *=* *0.002, respectively), while the differences were not significant for *S. glauca* and *S. salsa*.

**Table 3:** Results of standardized major axis regression (SMA) analysis of pairwise combinations of traits for each species in different saline–sodic soils

Y and X	*Chenopodium acuminatum*				*Chenopodium stenophyllum*				*Suaeda glauca*				*Suaeda salsa*			
	*P1*	*P2*	*P3*	*P4*	*P1*	*P2*	*P3*	*P4*	*P1*	*P2*	*P3*	*P4*	*P1*	*P2*	*P3*	*P4*
Plant height vs. stem diameter	***	*	Ns	***	***	*	ns	***	***	*	***	***	***	ns	ns	***
Plant height vs. root length	***	ns	***	***	***	**	***	***	**	ns	***	***	ns	ns	**	***
Root length vs. root diameter	***	ns	***	***	***	ns	***	***	ns	ns	***	***	*	ns	*	***
Root biomass vs. shoot biomass	***	***	***	***	***	**	***	***	***	ns	ns	***	***	ns	ns	***
Stem biomass vs. nonstem biomass	***	ns	***	***	***	ns	ns	***	***	ns	***	***	***	ns	**	***
Leaf biomass vs. nonleaf biomass	***	ns	***	***	***	ns	**	***	***	ns	***	***	***	ns	ns	***
Root biomass vs. leaf biomass	***	ns	***	***	***	*	***	***	***	ns	***	***	***	ns	ns	***

Note

*P1* is the test of the SMA regression for each relationship. *P2*,* P3*, and *P4* are tests of the shifts among soil treatments in terms of slope and elevation and along common slopes, respectively. ns, *p *>* *0.05; *, 0.05 ≥ *p *>* *0.01; **, 0.01 ≥ *p *>* *0.001; and ***, *p *<* *0.001.

## 4 DISCUSSION

### 4.1 Growth difference among four species in response to soil salinity–sodicity

Our study found that soil salinity–sodicity greatly inhibited plant growth, such as plant height, biomass, and stem diameter, which was consistent with the findings of others (Hooks et al., 2018; Javid et al., 2012; Kuehny & Morales, 1998; Shi & Sheng, 2005). When the soil salinity–sodicity increased from the non‐ to high level, the two species with low tolerance rapidly decreased all their growth traits and root mass ratio, possibly due to their higher threshold levels that induce osmotic effects and ionic imbalance in plant tissues (Ahmad, Ghafoor, Akhtar, & Khan, 2013; Maas, 1987), while the two highly tolerant species had the ability to maintain higher growth traits and a higher root mass ratio than the two *Chenopodium* species. Both *Suaeda* species are halophytes, which possibly have a distinctive physiological response to soil salinity or sodicity, such as strong osmotic adjustment and vacuolar compartmentation of toxic ions (Ravindran, Venkatesan, Balakrishnan, Chellappan, & Balasubramanian, 2007; Yang et al., 2007).

### 4.2 Plasticity in species with low and high saline–sodic tolerance

Plants have a remarkable capacity to adjust their morphological and physiological traits in response to abiotic conditions through acclimation or, more broadly, phenotypic plasticity (Bhattarai et al., 2017; Delagrange, Messier, Lechowicz, & Dizengremel, 2004; Sultan, 2000; Valladares & Niinemets, 2008). For the growth and allocation traits explored in this study, the species with saline–sodic tolerance exhibited higher phenotypic plasticity in response to soil salinity–sodicity than the highly tolerant species. Our finding was in agreement with previous studies on shade stress where species with low tolerance exhibited greater plasticity than highly tolerant species (Chmura et al., 2017; Portsmuth & Niinemets, 2007; Valladares & Niinemets, 2008). The lower plasticity of highly saline–sodic tolerant species suggests that tolerance is associated with phenotypic stability and a conservative resource‐use strategy even when resources are temporarily abundant (Balaguer et al., 2001; Valladares et al., 2002). Our results show that saline–sodic tolerant species exhibit lower phenotypic plasticity than saline–sodic sensitive species. This suggests that phenotypic plasticity is negatively correlated with fitness‐related traits and is inconsistent with previous findings that phenotypic plasticity can be regarded as an index of species sensitivity to soil salinity–sodicity (Couso & Fernández, 2012).

### 4.3 Growth plasticity versus allocation plasticity

Recent debates have focused on whether growth or allocation plasticity is more dominant in response to abiotic stress (Chmura et al., 2017; Curt et al., 2005; Fujii & Kasuya, 2008; Kramer‐Walter & Laughlin, 2017; Valladares et al., 2005). Previous studies showed that allocation traits vary little under biotic or abiotic stress (Curt et al., 2005; Kaelke, Kruger, & Reich, 2001; Reich et al., 1998). This study found that the highly tolerant species exhibited less allocation plasticity compared with growth plasticity, while the species with low tolerance exhibited higher growth and allocation plasticity. This implies that the highly tolerant species (*S. glauca* and *S. salsa*) have the ability to adapt to abiotic stress by adjusting their growth traits, which is a more conservative strategy in terms of biomass allocation compared with the species with low tolerance (*C. acuminatum* and *C. stenophyllum*). Our findings that growth traits are more sensitive than biomass allocation are more applicable to the highly tolerant species than the species with low tolerance.

### 4.4 “Apparent” versus “real” plasticity

Our results show that growth and allocation plasticity are a result of ontogenetic changes or environmental factors. Across 28 pairs of allometric relationships (Table 2), only 25% of the plasticity was “real” plasticity, that is, resulting from the soil salinity–sodicity (heterogenetic slope), while 75% of the plasticity was “apparent” plasticity, that is, resulting from the allometric coefficient (heterogenetic elevation) and the plant size (shifting along a common slope and elevation). The allometric results suggest that the phenotypic plasticity was mostly due to differences in individual size in the different saline–sodic soils (Weiner, 2004). The soil salinity–sodicity alters biochemical processes within the plant, which in turn affect plant development, typically resulting in varying plant size. In response to the soil salinity–sodicity, all species modified their size, consequently resulting in phenotypic plasticity. Soil salinity–sodicity only affects 25% of the allometric relationships, especially plant height versus stem diameter and root versus shoot mass. The allometric relationship between plant height and stem diameter has been theoretically and empirically evaluated in previous studies (Enquist, 2002; Li, Weiner, Zhou, Huang, & Sheng, 2013; Thomas, Martin, & Mycroft, 2015). The phenotypic plasticity for most traits in this study was ontogenetic, that is, “apparent” plasticity, but for plant height versus stem diameter and root versus shoot biomass, the species with low tolerance adjusted both their plant size and growth pattern in response to soil salinity–sodicity. This suggests that the plasticity in growth traits and biomass allocation is largely ontogenetic.

## 5 CONCLUSIONS

In summary, soil salinity–sodicity inhibited plant growth in species with both low and high tolerance and had more significant negative effects on root allocation in species with low tolerance than highly tolerant species. Growth and biomass allocation plasticity were found to be negatively correlated with saline–sodic tolerance. For highly saline–sodic tolerant species, growth traits are more sensitive to salinity–sodicity than allocation traits, while both growth and allocation traits are sensitive to soil salinity–sodicity in species with low tolerance. Thus, the species with low saline–sodic tolerance have greater plasticity in response to environmental variation, while the highly saline–sodic tolerant species maintained high fitness in unfavorable environments. Our results show that the plastic response could be either “apparent” (75%) or “real” (25%). To elucidate the actual mechanistic differences among species with different soil salinity–sodicity tolerances, we therefore strongly recommend that future studies should address whether phenotypic plasticity is “apparent” or “real” plasticity.

## CONFLICT OF INTEREST

None declared.

## AUTHOR CONTRIBUTIONS

YH and DZ designed the study; YH collected the data; YH, GF, and JP analyzed the data. YH and JP wrote the manuscript, and all authors contributed to the final version of the paper. All authors read and approved the final manuscript.
