# Supplementary material for: Phenotypic plasticity of four Chenopodiaceae species with contrasting saline–sodic tolerance in response to increased salinity–sodicity
Source: Ecol Evol. 2019 Feb 10;9(4):1545–53. doi: 10.1002/ece3.4515 (PMC6392380; doi:10.1002/ece3.4515)
Supplement: Supplementary file 1 [file ECE3-9-1545-s001.docx]

Table S1 Plant traits under different saline-sodic soil treatments. The soil treatments included non- (N), low (L), moderate (M), high (H) and extreme (E) saline-sodic soil. Means for each individual trait with the same letters are not significantly different between treatments within each trait (total is 18 mean values of all species) according to Duncan's multiple range tests. NA means the species can not survive.

| Traits | Soil | *C. acuminatum* | *C. stenophyllum* | *S. glauca* | *S. salsa* |
| --- | --- | --- | --- | --- | --- |
| Plant height | N | 61.5 ± 2.6 de | 109.1 ± 3.8 a | 82.5 ± 3.5 bc | 42.1 ± 2.1 ghij |
|  | L | 44.2 ± 2.1 ghi | 69.8 ± 3.2 cd | 92.9 ± 4.9 b | 36.7 ± 1.3 ijk |
|  | M | 29.8 ± 1.4 jk | 46.3 ± 3.0 fghi | 47.4 ± 2.7 efghi | 25.8 ± 1.3 k |
|  | H | 28.7 ± 1.7 jk | 60.4 ± 3.9 def | 56.0 ± 3.0 defg | 33.3 ± 1.7 ijk |
|  | E | NA | NA | 51.1 ± 2.2 efgh | 38.0 ± 1.2 hijk |
| Total biomass | N | 12.3 ± 3.1 b | 20.9 ± 3.5 a | 13.2 ± 3.0 b | 12.3 ± 1.8 b |
|  | L | 4.3 ± 0.7 cd | 6.3 ± 1.0 c | 16.5 ± 3.6 ab | 6.1 ± 0.7 c |
|  | M | 0.6 ± 0.1 d | 1.9 ± 0.3 cd | 3.4 ± 1.0 cd | 2.1 ± 0.3 cd |
|  | H | 1.1 ± 0.1 cd | 4.3 ± 1.2 cd | 5.4 ± 0.6 cd | 5.7 ± 1.3 cd |
|  | E | NA | NA | 3.7 ± 0.9 cd | 6.0 ± 0.7 c |
| Stem diameter | N | 0.49 ± 0.04 bc | 0.61 ± 0.04 a | 0.52 ± 0.04 ab | 0.51 ± 0.03 bc |
|  | L | 0.31 ± 0.02 efgh | 0.35 ± 0.02 def | 0.54 ± 0.04 ab | 0.42 ± 0.02 cd |
|  | M | 0.20 ± 0.01 i | 0.24 ± 0.02 ghi | 0.30 ± 0.03 efgh | 0.29 ± 0.02 fghi |
|  | H | 0.22 ± 0.01 hi | 0.28 ± 0.03 fghi | 0.39 ± 0.02 de | 0.37 ± 0.03 def |
|  | E | NA | NA | 0.31 ± 0.03 efg | 0.41 ± 0.02 cd |
| Root length | N | 19.0 ± 1.6 cd | 23.4 ± 2.1 b | 24.1 ± 2.3 b | 13.9 ± 0.8 efgh |
|  | L | 25.2 ± 1.3 ab | 29.0 ± 1.6 a | 22.1 ± 1.9 bc | 14.1 ± 0.6 efgh |
|  | M | 14.8 ± 1.2 defgh | 13.7 ± 1.1 efgh | 16.9 ± 0.7 de | 12.5 ± 0.7 fgh |
|  | H | 10.6 ± 0.9 h | 15.4 ± 1.1 defg | 13.2 ± 0.9 efgh | 13.7 ± 1.3 efgh |
|  | E | NA | NA | 16.8 ± 1.2 def | 12.0 ± 0.9 gh |
| Root diameter | N | 0.59 ± 0.05 b | 0.79 ± 0.07 a | 0.54 ± 0.06 bc | 0.58 ± 0.04 b |
|  | L | 0.34 ± 0.03 efg | 0.37 ± 0.02 def | 0.57 ± 0.06 bc | 0.45 ± 0.02 cde |
|  | M | 0.18 ± 0.01 h | 0.25 ± 0.02 fgh | 0.30 ± 0.03 fgh | 0.33 ± 0.03 efg |
|  | H | 0.23 ± 0.01 gh | 0.33 ± 0.04 efg | 0.44 ± 0.03 cde | 0.44 ± 0.04 cde |
|  | E | NA | NA | 0.33 ± 0.03 efg | 0.50 ± 0.03 bcd |
| Height:stem diameter ratio | N | 136.0 ± 9.0 g | 190.3 ± 11.8 abc | 170.6 ± 10.7 bcdef | 91.3 ± 9.3 h |
|  | L | 151.8 ± 7.9 defg | 203.0 ± 6.7 ab | 181.3 ± 8.2 bcd | 91.4 ± 5.4 h |
|  | M | 155.3 ± 6.2 defg | 200.3 ± 11.1 ab | 164.6 ± 7.0 cdefg | 94.6 ± 4.2 h |
|  | H | 136.4 ± 8.1 fg | 221.9 ± 6.8 a | 145.3 ± 4.4 efg | 98.6 ± 8.2 h |
|  | E | NA | NA | 173.0 ± 7.5 bcde | 96.6 ± 6.2 h |
| Height:root length ratio | N | 3.7 ± 0.4 bcde | 5.6 ± 0.7 a | 3.9 ± 0.4 bcd | 3.3 ± 0.2 defg |
|  | L | 1.8 ± 0.1 i | 2.5 ± 0.1 fghi | 4.7 ± 0.4 ab | 2.7 ± 0.2 efghi |
|  | M | 2.2 ± 0.2 ghi | 3.9 ± 0.5 bcd | 3.0 ± 0.3 defgh | 2.1 ± 0.1 hi |
|  | H | 3.0 ± 0.3 defgh | 4.4 ± 0.5 bc | 4.7 ± 0.5 ab | 2.7 ± 0.2 efghi |
|  | E | NA | NA | 3.2 ± 0.2 defgh | 3.4 ± 0.2 cdef |
| Root length:diameter ratio | N | 35.5 ± 3.6 efg | 32.4 ± 2.8 efg | 49.5 ± 5.3 cde | 26.2 ± 2.4 fg |
|  | L | 82.4 ± 6.2 ab | 81.3 ± 5.4 ab | 44.0 ± 5.0 def | 32.7 ± 2.1 efg |
|  | M | 86.2 ± 8.4 a | 58.5 ± 5.5 cd | 65.8 ± 5.9 bc | 41.7 ± 3.3 defg |
|  | H | 47.7 ± 4.4 de | 58.1 ± 7.4 cd | 32.2 ± 2.7 efg | 33.5 ± 3.2 efg |
|  | E | NA | NA | 53.5 ± 3.4 cd | 25.7 ± 2.3 g |
| Root mass ratio | N | 0.13 ± 0.02 ab | 0.13 ± 0.02 a | 0.13 ± 0.02 ab | 0.08 ± 0.01 fg |
|  | L | 0.14 ± 0.01 a | 0.11 ± 0.01 abcd | 0.11 ± 0.02 abcdef | 0.08 ± 0.00 efg |
|  | M | 0.11 ± 0.01 abcd | 0.10 ± 0.01 bcdefg | 0.11 ± 0.01 abcde | 0.08 ± 0.01 defg |
|  | H | 0.07 ± 0.00 g | 0.09 ± 0.01 cdefg | 0.12 ± 0.01 abc | 0.09 ± 0.01 cdefg |
|  | E | NA | NA | 0.10 ± 0.01 bcdefg | 0.13 ± 0.01 a |
| Leaf mass ratio | N | 0.05 ± 0.01 d | 0.06 ± 0.01 d | 0.13 ± 0.02 cd | 0.39 ± 0.01 ab |
|  | L | 0.04 ± 0.01 d | 0.07 ± 0.01 d | 0.11 ± 0.01 d | 0.39 ± 0.01 ab |
|  | M | 0.05 ± 0.01 d | 0.09 ± 0.01 d | 0.22 ± 0.02 c | 0.47 ± 0.02 a |
|  | H | 0.09 ± 0.01 d | 0.07 ± 0.01 d | 0.11 ± 0.01 d | 0.42 ± 0.01 ab |
|  | E | NA | NA | 0.22 ± 0.02 c | 0.36 ± 0.01 b |
| Stem mass ratio | N | 0.29 ± 0.01 i | 0.39 ± 0.01 efgh | 0.51 ± 0.02 abc | 0.53 ± 0.01 ab |
|  | L | 0.35 ± 0.02 hi | 0.37 ± 0.01 gh | 0.55 ± 0.01 a | 0.52 ± 0.01 ab |
|  | M | 0.37 ± 0.02 fgh | 0.41 ± 0.02 efgh | 0.4 0± 0.02 efgh | 0.43 ± 0.02 def |
|  | H | 0.30 ± 0.01 i | 0.37 ± 0.01 fgh | 0.45 ± 0.02 cde | 0.48 ± 0.01 bcd |
|  | E | NA | NA | 0.43 ± 0.02 defg | 0.50 ± 0.01 abc |
| Shoot:root mass ratio | N | 8.5 ± 0.9 fg | 8.4 ± 0.9 fg | 8.7 ± 0.9 efg | 13.4 ± 0.9 ab |
|  | L | 7.3 ± 0.7 g | 8.8 ± 0.7 efg | 10.6 ± 1.1 bcdef | 11.8 ± 0.5 abcd |
|  | M | 8.5 ± 0.6 fg | 10.1 ± 0.8 cdef | 8.9 ± 0.7 efg | 12.0 ± 0.9 abc |
|  | H | 13.7 ± 0.8 a | 11.5 ± 0.9 abcde | 9.2 ± 0.8 defg | 10.8 ± 0.7 bcdef |
|  | E | NA | NA | 10.5 ± 0.8 cdef | 8.8 ± 1.8 efg |
| Root:leaf mass ratio | N | 7.64 ± 4.20 a | 5.52 ± 1.89 ab | 1.73 ± 0.38 cd | 0.20 ± 0.02 d |
|  | L | 7.10 ± 1.57 a | 3.24 ± 1.24 bcd | 1.16 ± 0.21 cd | 0.21 ± 0.01 d |
|  | M | 4.36 ± 0.78 abc | 1.48 ± 0.21 cd | 0.58 ± 0.07 d | 0.18 ± 0.01 d |
|  | H | 1.02 ± 0.17 cd | 2.12 ± 0.43 bcd | 1.92 ± 0.66 cd | 0.22 ± 0.02 d |
|  | E | NA | NA | 0.54 ± 0.06 d | 0.38 ± 0.04 d |
